# Identification of a *de novo* mutation of the *FOXG1* gene and comprehensive analysis for molecular factors in Chinese FOXG1-related encephalopathies

**DOI:** 10.3389/fnmol.2022.1039990

**Published:** 2022-12-07

**Authors:** Guanting Lu, Yan Zhang, Huiyun Xia, Xiaoyan He, Pei Xu, Lianying Wu, Ding Li, Liya Ma, Jin Wu, Qiongling Peng

**Affiliations:** ^1^Laboratory of Translational Medicine Research, Department of Pathology, Deyang People's Hospital, Deyang, China; ^2^Key Laboratory of Tumor Molecular Research of Deyang, Deyang, China; ^3^Department of Obstetrics and Gynecology, Strategic Support Force Medical Center, Beijing, China; ^4^Department of Child Healthcare, Shenzhen Baoan Women's and Children's Hospital, Jinan University, Shenzhen, China

**Keywords:** Rett syndrome, FOXG1-related encephalopathy, FOXG1, PRKD1, haploinsufficiency, intergenic regulatory elements

## Abstract

**Background:**

FOXG1-related encephalopathy, also known as FOXG1 syndrome or FOXG1-related disorder, affects most aspects of development and causes microcephaly and brain malformations. This syndrome was previously considered to be the congenital variant of Rett syndrome. The abnormal function or expression of FOXG1, caused by intragenic mutations, microdeletions or microduplications, was considered to be crucial pathological factor for this disorder. Currently, most of the FOXG1-related encephalopathies have been identified in Europeans and North Americans, and relatively few Chinese cases were reported.

**Methods:**

Array-Comparative Genomic Hybridization (Array-CGH) and whole-exome sequencing (WES) were carried out for the proband and her parent to detect pathogenic variants.

**Results:**

A *de novo* nonsense mutation (c.385G>T, p.Glu129Ter) of FOXG1 was identified in a female child in a cohort of 73 Chinese children with neurodevelopmental disorders/intellectual disorders (NDDs/IDs). In order to have a comprehensive view of FOXG1-related encephalopathy in China, relevant published reports were browsed and twelve cases with mutations in FOXG1 or copy number variants (CNVs) involving FOXG1 gene were involved in the analysis eventually. Feeding difficulties, seizures, delayed speech, corpus callosum hypoplasia and underdevelopment of frontal and temporal lobes occurred in almost all cases. Out of the 12 cases, eight patients (66.67%) had single-nucleotide mutations of FOXG1 gene and four patients (33.33%) had CNVs involving FOXG1 (3 microdeletions and 1 microduplication). The expression of FOXG1 could also be potentially disturbed by deletions of several brain-active regulatory elements located in intergenic FOXG1-PRKD1 region. Further analysis indicated that PRKD1 might be a cooperating factor to regulate the expression of FOXG1, MECP2 and CDKL5 to contribute the RTT/RTT-like disorders.

**Discussion:**

This re-analysis would broaden the existed knowledge about the molecular etiology and be helpful for diagnosis, treatment, and gene therapy of FOXG1-related disorders in the future.

## Introduction

Rett syndrome (RTT; OMIM#312750) is a severe syndromic disorder that affects almost exclusively females with a prevalence of 1/10,000 under an X-linked dominant (XLD) mode of inheritance (Grillo et al., 2012). The characterized clinical phenotypes include arrested growth at an early stage (usually between 6 months and 18 months after birth), loss of speech, withdrawal of acquired skills, hand stereotypies, microcephaly, seizures, and intellectual disability (Moog et al., 2003).

It has been reported that mutations of the Xq28-localized *MECP2* gene are responsible for about 90% of RTT cases. Except for *MECP2, CDKL5* (Zhu and Xiong, 2019) and *FOXG1* (Byun et al., 2015) are two other well-known RTT causal genes. Recently, *STXBP1* (Cogliati et al., 2019), *KIF1A* (Wang et al., 2019), *GRIN1* (Wang et al., 2019), *NTNG1* (Borg et al., 2005; Archer et al., 2006; Nectoux et al., 2007; Aldosary et al., 2020), *NTNG2* (Heimer et al., 2020), *MEF2C* (Wang et al., 2018; Anitha et al., 2022), *SATB2* (Lee et al., 2016), and *WDR45* (Hoffjan et al., 2016; Kulikovskaja et al., 2018) have also been implicated as genetic factors of RTT or RTT-like syndromes. According to the molecular assays for two large cohorts recruited 486 Chinese patients with RTT, *MECP2* accounted for 83.74% (407/486) and *CDKL5* for 0.82% of the cases. No mutations of *FOXG1* were detected (Li et al., 2007; Zhang et al., 2012). In 2017, four *de novo FOXG1* mutations were first reported in a cohort of 451 Chinese patients with RTT or RTT-like disorders (Zhang et al., 2017). Except for intragenic mutations, CNVs containing the *FOXG1* gene were also reported in Chinese patients with neurodevelopmental disorders (Wang et al., 2017; Li et al., 2021).

Previously, patients carrying pathogenic *FOXG1* mutations were initially diagnosed as a congenital variant of the RTT syndrome (OMIM#613454), for having global development delay and disease onset from early infancy (before 6 months of age) and seizure onset after 3 months of birth (Ariani et al., 2008; Jacob et al., 2009). However, with the accumulation of clinical phenotypes associated with FOXG1 mutations, the patients generally lacked social eye contact, faced more severe sleep difficulty, and experienced difficulty in the postnatal development of language and ambulation (Papandreou et al., 2016; Mitter et al., 2018; Vegas et al., 2018). More importantly, the patients also lacked obvious regression of required psychomotor abilities as observed in RTT (Caporali et al., 2018). Therefore, the spectrum associated with FOXG1 mutations has been considered to be a separate clinical entity and termed “FOXG1-related encephalopathy,” (Wong et al., 2019b) “FOXG1-related disorder,” (McMahon et al., 2015) or “FOXG1-related syndrome” (Wong et al., 2019a).

Recently, a *de novo* non-sense mutation (c.385G>T, p.Glu129Ter) of the *FOXG1* gene was identified in a Chinese female patient with neurodevelopmental disorders/intellectual disorders (NDD/IDs). Currently, most of the patients of FOXG1-related encephalopathy are of European and North American origins, and only 12 Chinese cases have been identified so far. In the current project, a comprehensive reanalysis of the genotypes of *FOXG1* was carried out for the Chinese FOXG1-related encephalopathies. Four main types of underlying molecular etiologies for this disorder were categorized, such as intragenic mutations, CNVs containing *FOXG1*, CNVs containing the intergenic region *FOXG1-PRKD1*, and the contributing gene *PRKD1*.

## Materials and methods

### Sample collection

This study was conducted in accordance with the Code of Ethics of the World Medical Association (Declaration of Helsinki) for experiments involving humans. This study was approved by the Ethical Committee of the Shenzhen Bao'an Women's and Children's Hospital (LLSC-2022-02-05-16-KS). Written informed consent was obtained from the parent. Peripheral venous blood was collected from the infant and her parent.

### Array-comparative genomic hybridization

Genomic DNA was extracted using the TIANamp Blood DNA Kit (DP348, TianGen Biotech, Beijing, China) according to the manufacturer's instructions. Genomic aberrations were detected by array-CGH using the Fetal DNA Chip (version 1.2) designed by The Chinese University of Hong Kong (CUHK) (Leung et al., 2011; Huang et al., 2014). Procedures of array-CGH were conducted according to the public Agilent protocol (Agilent Oligonucleotide Array-Based CGH for Genomic DNA Analysis, version 3.5). Briefly, hybridized slides were scanned with SureScan High-Resolution Microarray Scanner (G2505B, Agilent Technologies, Santa Clara, CA, USA), and the image data were extracted and converted to text files using the Agilent Feature Extraction software (version 10.5.1.1). The data were graphed and analyzed using the Agilent CGH Analytics software. Only duplications or deletions that were covered by at least three consecutive probes on the Fetal DNA Chip were considered.

### Trio-whole exome sequencing

Whole exome sequencing for the trio (Trio-WES) was conducted by the Illumina HiSeq 2500 platform (Illumina, San Diego, CA, United States) according to our previous reports (Lu et al., 2021; Peng et al., 2022). Briefly, 1 mg of sheared DNA was ligated with adaptors and then amplified by PCR. The amplified fragments were hybridized and captured with xGen Exome Research Panel v2.0 (Integrated DNA Technologies, Coralville, IA, USA) according to the manufacturer's protocol. The captured products were amplified, purified, and quantified using an Agilent Bioanalyzer 2100 (Agilent Technologies, Santa Clara, CA, USA). Finally, the established libraries were sequenced on the Illumina HiSeq 2500 platform and the NextSeq CN500 platform (Berry Genomics, Beijing, China) for paired-end sequencing.

The sequencing reads were aligned against the human reference genome (hg19/GRCh37) using the BWA software (version 0.7.10) (Li and Durbin, 2010). The Verita Trekker^®^ Variants Detection System (version 2.0, Berry Genomics, Beijing, China) was used for variant calling. The annotation and interpretation of the variants were conducted using Enliven Variants Annotation Interpretation System (Berry Genomics, Beijing, China) (Yang et al., 2019). A position was called heterozygous if 25% or more of the reads identify the minor allele. The retained variants for subsequent interpretation should have a minor allele frequency (MAF) <1% in 1000 Genomes Project, Exome Aggregation Consortium (ExAC) (Lek et al., 2016), NHLBI GO Exome Sequencing Project (ESP) (Fu et al., 2013), Genome Aggregation Database (gnomAD), NHLBI Trans-Omics for Precision Medicine (TOPMed) (Taliun et al., 2021). We also removed non-functional variants such as synonymous mutation and non-coding region mutation. According to the criteria for interpretation of genetic variants proposed by American College of Medical Genetics and Genomics (ACMG) guidelines, the annotated variants could be categorized into five classes, namely, “pathogenic,” “likely pathogenic,” “uncertain significance,” “likely benign,” and “benign” (Richards et al., 2015). The candidate variants were confirmed by Sanger sequencing.

### Compilation of Chinese patients with FOXG1-related disorder

Using “FOXG1”, “Forkhead box G1”, “14q12”, “Rett syndrome”, “RTT”, “Rett”, “Intellectual disability”, “Developmental delay”, “Chinese”, and “China” as keywords to search the English-written articles in NCBI PubMed (https://pubmed.ncbi.nlm.nih.gov/), and Chinese-written articles in WANFANG DATA (https://c.wanfangdata.com.cn/periodical), Chinese National Knowledge Infrastructure (CNKI) (https://www.cnki.net/), and VIP (http://www.cqvip.com/) periodical databases. Twenty-one relative articles were obtained from public periodical databases. After removing duplicates and reviews, eight articles (four English and four Chinese) were selected for subsequent analysis. After careful evaluation, one family was reported two times due to seeking medical consultations at different hospitals. Totally, six articles were qualified. The workflow chart was depicted in Supplementary Figure S1. Twelve cases with *FOXG1* mutations or CNVs involving the *FOXG1* gene were compiled for subsequent analysis.

### Molecular analysis of FOXG1-related Rett syndrome

To have a comprehensive analysis for mutations of the *FOXG1* gene, single nucleotide mutations, microduplications, and microdeletions were extracted from NCBI ClinVar, DECIPHER (Firth et al., 2009), ClinGen (the Clinical Genome Resource) (Rehm et al., 2015), copy number variants from 29,083 cases (nstd100) and 15,767 cases (nstd54) of Developmental Delay and Intellectual Disability (DD/ID) (Cooper et al., 2011; Coe et al., 2014), and Database of Genomic Variants (DGV) (MacDonald et al., 2014). The CNVs were mapped against the human genome (hg19) by the UCSC genome browser. The protein sequences of FOXG1 were downloaded from the NCBI gene track (https://www.ncbi.nlm.nih.gov/gene) and aligned with the integrated CLUSTALW tool of MEGA (version 11.0.8) under default settings. The protein structures of FOXG1 with Ser197Ile (S197I) and Asn232Tyr (N232Y) mutations were predicted with AlphaFold2. As for protein-coding genes, pHI (the probability of being a haploinsufficient gene) and pAD (the probability of being autosomal dominant) were analyzed by DECIPHER and DOMINO (https://wwwfbm.unil.ch/domino/index.php), respectively. The genomic conservation evolution analysis was carried out by the ECR browser (https://ecrbrowser.dcode.org/). The Hi-C (high-through chromosome conformation capture) data for seven human cell lines (namely, GM12878, K562, KBM7, HMEC, HUVEC, IMR90, and NHEK) were downloaded from gene expression omnibus (GEO) (https://www.ncbi.nlm.nih.gov/geo/query/acc.cgi?acc=GSE63525).

## Results

### Clinical features of our patient with a *FOXG1* mutation

**Figure 1:**
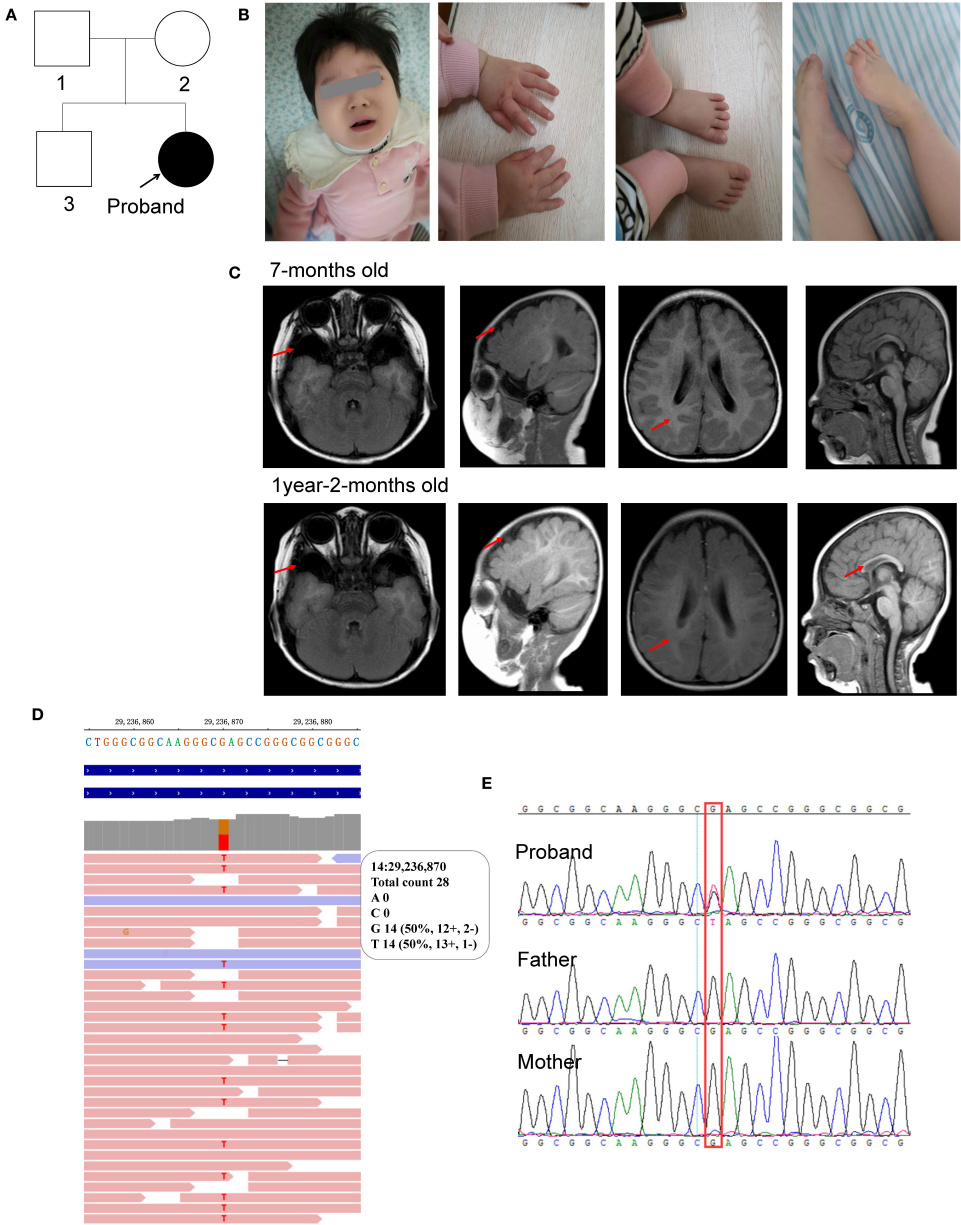
Characterization of the clinical features of the proband. **(A)** Pedigree of the proband; **(B)** photos of the facial, hands, and foot; **(C)** brain MRI scan taken at 7-months old and 1-year-2-months old; **(D)** WES identified c.385G>T in the proband; **(E)** sanger sequencing. The red arrow represents the abnormal region.

This patient was a 1 year and 11 months old female infant who was referred to our department because of early-onset delay of psychomotor development. The proband was delivered uneventfully at full-term to a 34-year-old mother by cesarean section due to breech position in 2019. Her birth weight was 3.09 kg. Her mother accepted all of the regular inspections as required and no abnormalities were found during her pregnancy. She was the second child of a non-consanguineous couple. Her 5-year-old brother had a normal developmental trajectory (Figure 1A). The patient was found obvious developmental delay after birth and was diagnosed with developmental delay till she was 7 months old. She had typical clinical phenotypes involving impaired social interaction, lack of speech development, delayed motor development, stereotypic movements of hands, hypotonia, bruxism while awake, sleep rhythm disorder, and seizure (Figure 1B).

For physical development, her height was 77 cm (<P3) and her weight was 10 kg (P18), which was suggested to be short stature. Her occipitofrontal head circumference (OFC) was 42 cm (<P3), referred to be microcephalus. For psychomotor development, she could not gain full head control and smooth roll over. She could neither sit nor stand without assistance. She could not develop hand grasping with a specific purpose. Excessive unconscious hand movements were observed, such as shaking and flapping. Eye contact was very few. A lack of speech development was observed. Hypotonia was observed after birth. Feeding difficulties were seen after adding semisolid food. Sleeping disturbance has been observed soon after birth and lasted till now. She also had bruxism while awake.

When she was 1 year and 6 months old, the seizure was first observed with a sudden loss of consciousness, eye gaze, and limb spasms that spontaneously resolved after 1–3 min. An episode of seizure occurred almost daily. She was diagnosed with generalized tonic-clonic seizures and began to receive antiseizure therapy 2 months later with oral antiepileptic drugs, such as topiramate, Depakin, Topamax, and i.v. drip of adrenocorticotropic hormone (ACTH). The seizures were relieved but not completely controlled. At present, the seizures still occurred two or three times a month.

Her first regular electroencephalogram (EEG) at 7 months old was normal. Her 24-h video of EEG at 1 year and 8 months showed generalized temporal spike and spike-slow waves during the wakefulness and sleep stages. Her first brain magnetic resonance imaging (MRI) scan at 7 months old showed widened bilateral temporal extracerebral space (the widest spaces were 14 mm on the right and 19 mm on the left, respectively), small bilateral temporal lobes, and delayed myelination development (equivalent to 3–4 months). At the age of 1 year and 2 months, she took her second MRI scan. Except widened bilateral temporal extracerebral space, small bilateral temporal lobes, and delayed myelination development (equivalent to 6–7 months) as before, corpus callosum hypoplasia was also observed (Figure 1C).

She accepted examinations of neural electrophysiological examinations involving flash visually evoked potential (fVEP) and auditory brainstem response (ABR) when she was 9 months old. The fVEP showed bilateral prolonged baseline P100 and N145 latencies. Auditory brainstem response (ABR) showed bilateral prolonged latencies of waves I, III, and V. No abnormality of the urinary system was found by color Doppler ultrasound at 10 months old. DR X-ray film for hip joint anteroposterior and abducent projections at 1 year and 11 months old found no abnormalities.

Molecular analysis with whole exome sequencing identified a *de novo* non-sense mutation of FOXG1 (c.385G>T, rs1555321264, and 14:29236870) in the patient (Figure 1D) and further confirmed by Sanger sequencing (Figure 1E). The c.385G>T mutation generated a premature stop codon at position 129 (GAG) for glutamic acid (Glu, E) to (TAG, X) (p.Glu129Ter). This mutation was not been detected in any of the four public human genome projects, such as 1000 Genomes (*n* = 2,504), GO-ESP (*n* = 6,503), ExAC (*n* = 60,706), gnomAD Genomes (*n* = 15,708), gnomAD Exomes (*n* = 125,748), and TOPMED (*n* = 25,199,470) databases. According to the ACMG guideline, this mutation was classified to be pathogenic (PVS1+PM2+PP5).

Combined with characteristic clinical phenotypes, brain MRI, and molecular analysis, the child was diagnosed as a congenial variant of Rett syndrome, (OMIM#613454), which was also named FOXG1-related encephalopathy.

### Comprehensive analysis of 12 Chinese with FOXG1 mutations

**Figure 2:**
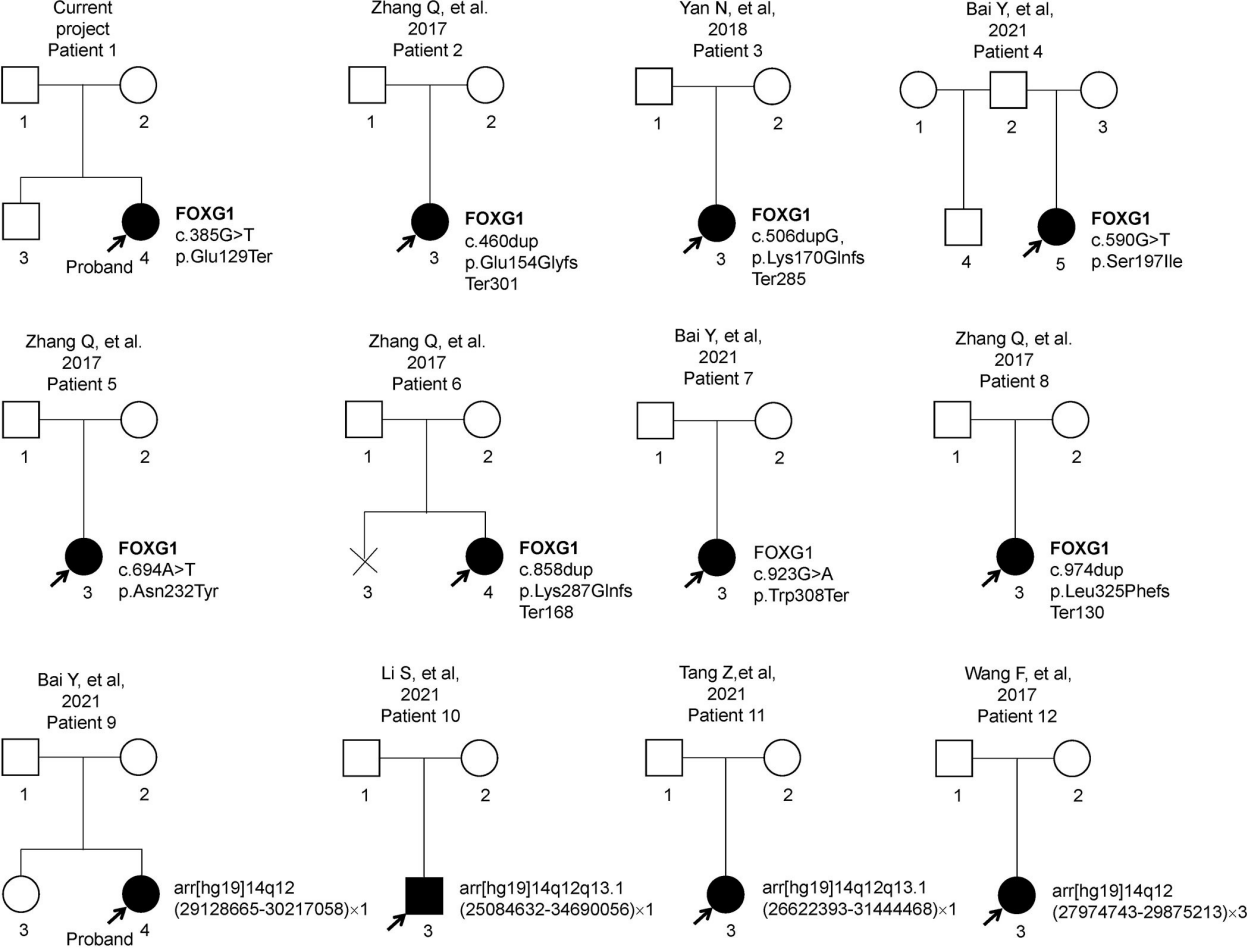
Pedigrees of 12 patients with FOXG1-related encephalopathy.

Previous articles have reported 11 individual Chinese cases with 11 *de novo* FOXG1 mutations. To have a comprehensive reanalysis of these mutations, the medical records and clinical phenotypes of all the patients (11 + 1) were carefully inquired and compiled (Table 1). Pedigrees of all 12 patients were depicted in Figure 2. The ranking of the pedigrees was based on the mutation's location on the FOXG1 protein.

**Table 1:** Characterizations of the 12 Chinese patients with FOXG1 mutations.

**General information**												
**Cases**	**Our project**	**Zhang et al. (2017)**	**Yang et al. (2019)**	**Bai et al. (2021)**	**Zhang et al. (2017)**	**Zhang et al. (2017)**	**Bai et al. (2021)**	**Zhang et al. (2017)**	**Bai et al. (2021)**	**Li et al. (2021)**	**Tang et al. (2021)**	**Wang et al. (2017)**
Patient No.	Patient 1	Patient 2	Patient 3	Patient 4	Patient 5	Patient 6	Patient 7	Patient 8	Patient 9	Patient 10	Patient 11	Patient 12
Sex	Female	Female	Female	Female	Female	Female	Female	Female	Female	Male	Female	Female
Age	1 y 11 m	1 y	2 y 5 m	1 y 6 m	4 y 6 m	1 y 8 m	2 y	2 y	2 y 6 m	8 d	3 y	9 y
Molecular analysis												
Locations (hg19)	14:29236870	14:29236945	14:29236986	14:29236986	14:29237179	14:29237343	14:29237408	14:29237457	14:29128665- 30217058	14:25084632- 34690056	14:26622393-31444468	14:28904992- 30805462
Genetic mutations	c.385G>T (p.Glu129Ter)	c.460dup (p.Glu154 GlyfsTer301)	c.506dup (p.Lys170 GlnfsTer285)	590G>T (p.Ser197Ile)	c.694A>T (p.Asn232Tyr)	c.858dup (p.Lys287 GlnfsTer168)	c.923G>A (p.Trp308Ter)	c.974dup (p.Leu325 PhefsTer130)	/	/	/	/
CMAs	/	/	/	/	/	/	/	/	arr[hg19]14q12 (29128665- 30217058)x1	arr[hg19]14 q12q13.1 (25084632- 34690056)x1	arr[hg19]14q12 (26622393- 31444468)x1	arr[hg19]14q12 (28904992- 30805462)x3
Consequence	Stopgain	Frameshift	Frameshift	Missense	Missense	Frameshift	Stopgain	Frameshift	Microdeletion	Microdeletion	Microdeletion	Microduplication
Karyotypes	46, XX	46, XX	46, XX	46, XX	46, XX	46, XX	46, XX	46, XX	46, XX del(14)(q12)	46, XY del(14)(q12q13.1)	46, XX del(14)(q12)	46, XX dup(14)(q12)
ACMG classification	Pathogenic PVS1+ PS2+PM2	Pathogenic PVS1+ PS2+PM2	Pathogenic PVS1+ PS2+PM2	Likely pathogenic M1+ PM2+PP2+ PP3+PP5	Likely pathogenic PS2+ PM2+PP3	Pathogenic PVS1+ PS2+PM2	Pathogenic PVS1+ PM2+PP5	Pathogenic PVS1+ PS2+PM2	/	/	/	/
dbSNP ID	rs1555321264	rs398124204	rs1452295073	/	rs786205486	/	/	/	/	/	/	/
Inheritance	*De novo*	*De novo*	*De novo*	*De novo*	*De novo*	*De novo*	*De novo*	*De novo*	*De novo*	*De novo*	*De novo*	*De novo*
Head circumstance (cm)	42	43	40.5	39	49	42	38.5	45.5	/	/	/	46
Microcephaly	Y	Y	Y	Y	N	Y	Y	Y	/	/	/	Y
Regression	N	N	Y	N	N	Y	N	N	Y	/	/	/
Bruxism	Y	Y, 12 m	/	/	Y, 12 m	Y, 19 m	/	N	/	/	/	/
Hypotonia	Y	Y	Y	Y	Y	Y	Y	Y	Y	Y, 8 d	/	Y
Stereotypic movements	Y	Y, 3 m	Y	Y	Y, 10 m	Y, 12 m	Y	Y, 8 m	Y	/	/	Y
Limited functional hand use	Y	Y	/	Y	Y	Y	Y	Y	Y	/	/	Y
Rising head (m)	Y,10 m	Y, 8 m	Y, 11 m	Y	Y, 5 m	Y, 7 m	Y	Y, 2 m	Y,9 m	/	/	Y, 36 m
Sitting	N	N	N	Y	Y, 12 m	N	Y	Y, 10 m	Y,11 m	/	/	Y, 48 m
Walking	N	N	N	N	N, standing with aid at 24 m	N	N	N	N	/	/	Y,72 m
Poor eye contact	Y	Y	Y	Y	Y	/	Y	Y	Y	/	Y	Y
Delayed speech	Y	Y	Y	Y	Y	Y	Y	Y	Y	/	Y	Y
Feeding difficulties	Y	Y	Y	/	Y	/	/	Y	/	Y, 8 d	/	Y
EEG abnormalities	Y	Y	Y	N	Y	Y	N	Y	N	/	Y	Y
Seizure onset time (m)	Y, 18 m	Y, 10 m	Y, 16 m	N	Y, 6 m	Y, 10.5 m	N	Y, 10.5 m	Y, 13–15 m	/	Y, ?	Y, 4 m
Seizure types	Generalized tonic-clonic	Partial	Generalized tonic-clonic	N	Partial	Partial	N	Partial	/	/	?	?
Seizure with cyanosis	N	Y	/	N	Y	Y	N	Y	Y	/	/	/
Sleep disturbances	Y	Y	Y	/	Y	N	/	Y	/	/	/	/
Corpus callosum hypoplasia	Y	Y	Y	N	Y	Y	N	Y	N	Y	/	Y
Delayed myelination or hypomyelination	Y	N	N	N	N	Y	N	N	N	/	/	/
Underdevelopment of frontal and temporal lobes	Y	Y	Y	Y	Y	Y	Y	Y, mild	Y	/	/	Y

After compiling the described clinical features of the 12 patients (Table 1), we noticed that 11 of them were female (91.67%) and only 1 male (8.33%). Since the clinical descriptions for patients 10 and 11 were scarce, they were removed for the subsequent clinical spectrum analysis. The head size development of eight patients (8/10) was lagging behind the standard of the same age and displayed a microcephaly phenotype. The regression was reported in three patients with the progressive disappearance of acquired language skills. Bruxism was also identified in four patients. As for abnormalities of the musculoskeletal system, hypotonia and stereotypic movements with limited functional hand use were reported in almost all the patients. In the early stages, no patients could walk or sit normally; however, with increasing age, six patients could sit without aid after 10 months. Patient 11 could walk without aid at 3 years old. As for the eyes, almost all the patients had poor eye contact and could not follow moving objects.

As for the features concerning the central nervous system (CNS), delayed speech, feeding difficulties, seizures, abnormal EEGs, and stereotypic movements were reported in most of the patients. As for seizures, the onset time was at or after 4 months, and in patient 1, it was at 18 months. Two types of seizures were reported in these patients, such as partial or generalized tonic-clonic. According to the reported MRI/CT results, corpus callosum hypoplasia and underdevelopment of the frontal and temporal lobes were seen in most of the patients. Delayed myelination or hypomyelination was reported in only two patients.

### Single-nucleotide mutations identified by next-generation sequencing

**Figure 3:**
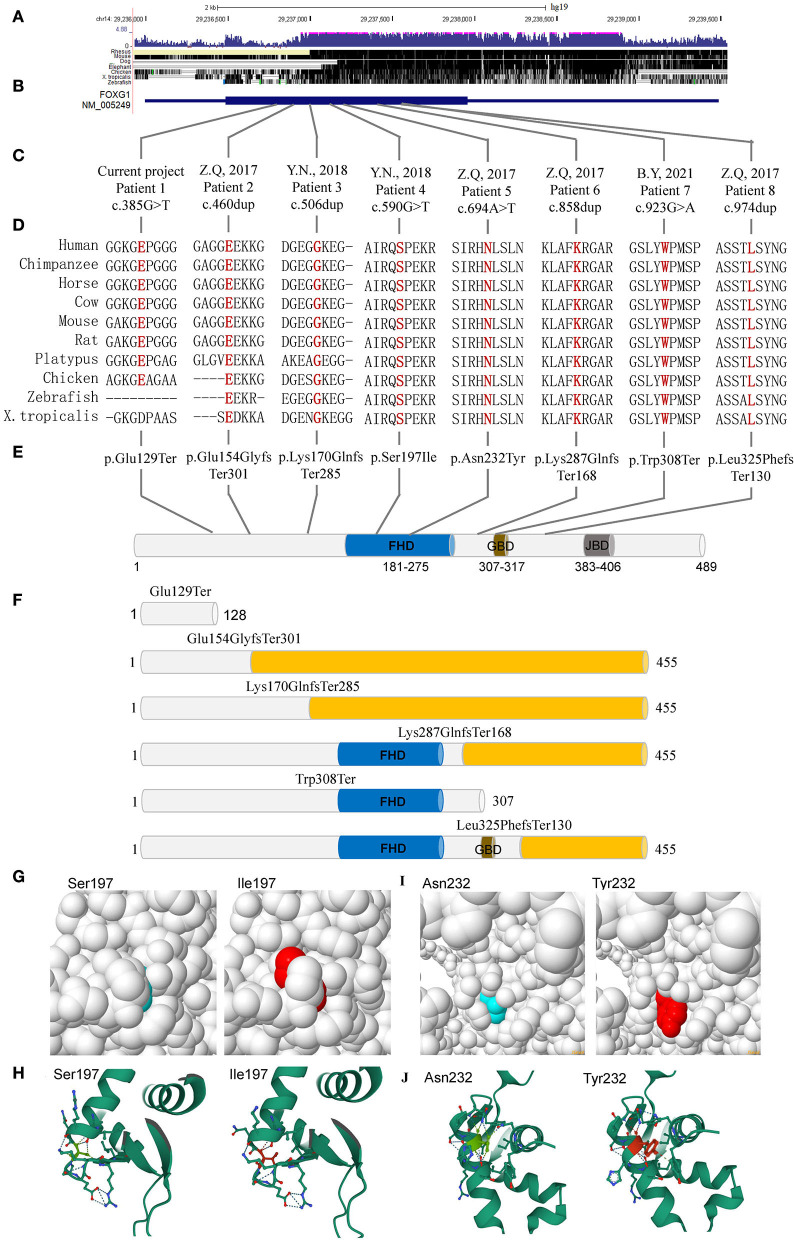
Single-nucleotide mutations of FOXG1 identified in Chinese patients. **(A)** Conservation of the genomic region; **(B)** Gene structure of FOXG1; **(C)** Nucleotide changes of the mutations; **(D)** Conservation analysis of the mutations; **(E)** Amino acid changing of the mutations; **(F)** Proteins containing truncating mutations. **(G)** Structure of FHD domain with Ser197; **(H)** Structure of FHD domain with Ile197; **(I)** Structure of FHD domain with N232; **(J)** Structure of FHD domain with Y232. FHD, DNA binding fork-head domain; GBD, Groucho-binding domain; JBD, JARID1B binding domain. The yellow cylinder represents the aberrant amino acids caused by frameshifting mutations.

The *FOXG1* gene was mapped to an evolutionarily conserved region (Figure 3A), including only one exon (Figure 3B). To date, there are eight heterozygous single-nucleotide mutations of *FOXG1* detected in Chinese patients with intellectual disability (ID). The details of these mutations were described in Table 1. For our patient (patient 1), the *de novo* nonsense mutation, c.385G>T (Figure 3C) generated a premature stop codon at position 129 (GAG) for glutamic acid (Glu, E) to (TAG, X) (p.Glu129Ter). As far as we know, this mutation has been detected in an individual with unknown diseases by GeneDx in 2017 and recruited in ClinVar. Besides, no pathogenic mutations were detected in other genes for RTT/RTT-like disorders. As for mutations in patients 2, 4, 5, and 6, all four mutations were insertions with one nucleotide, which caused frame-shifting of the original coding sequence (CDS) of *FOXG1* (Figures 3C–E). c.460dup (rs398124204 in patient 2) was reported more than 10 times in European patients (Bahi-Buisson et al., 2010; Van der Aa et al., 2010; Kortüm et al., 2011; Bean et al., 2013; Seltzer et al., 2014; Richards et al., 2015; Cellini et al., 2016; Nykamp et al., 2017; Mitter et al., 2018; Vegas et al., 2018) and only once in Chinese patients (Zhang et al., 2017). c.506dup (rs1450095073 in patient 3) and the other two frame-shift mutations (c.858dup in patent 5 and c.974dup in patient 6) had been submitted to the NCBI ClinVar database by GeneDx and reported in two Chinese patients with Rett syndrome (Zhang et al., 2017; Niu et al., 2020). Although the inserted locations of the mutations were varied, the CDSs containing these four frame-shifting mutations terminated at the same premature stop codon to produce a truncated protein with 455 amino acids (Figure 3F). According to the criteria of ACMG classification, these mutations were annotated as “pathogenic” (PVS1+PS2+PM2).

As for the two missense mutations (c.590G>T, p.Ser197Ile in patient 4 and c.694A>T, p.Asn232Tyr in patient 5), they were located in the DNA-binding forkhead domain (FHD). Both amino acids (such as Ser197 and Asn232) were strongly conserved (Figure 3D) during evolution and not detected in the known public genomic databases, such as 1000 Genome (*n* = 2,504), NHLBI GO-ESP (*n* = 6,503), ExAC (*n* = 60,706), gnomAD (*n* = 15,708), and TOPMED (*n* = 60,000). According to the criteria of ACMG classification, they were annotated as “likely pathogenic” (Table 1). For c.590G>T (p.Ser197Ile), it has been submitted to the ClinVar database by the Genetic Services Laboratory of the University of Chicago (Accession: SCV002069287.1) and identified in a 47-month-old patient (Mitter et al., 2018). Analyzed by Missense3D, the 197Ile substitution disrupts all side-chain/main-chain H-bonds formed by the buried Ser residue (RSA 2.3%) (CO197-Ser OG/CO194-Ile O, CO197-Ser OG/CO201-Arg O; CO200-Lys NZ/CO197Ser O, CO200Lys N/CO197Ser OG, CO201-Arg N/CO197Ser OG). This mutation also results in a switch from the buried Ser (RSA 2.3%) to exposed Ile (26.6%) (Figures 3G,H). The buried H-bond breakage and buried/exposed switch might disrupt the local structure of the PHD domain. For c.694A>T (p.Asn232Tyr), it has been submitted to the ClinVar database by the DGU-KFSHRC (Developmental Genetics Unit, King Faisal Specialist Hospital & Research Centre) and reported in a Chinese patient with RTT (Zhang et al., 2017). The side chain of Asn (N) is a small-sized amino carbonyl but a bulky p-hydroxyphenyl for Tyr (Y). Analyzed by Missense3D, this mutation led to a switch from a buried Asn (RSA 7.0%) to an exposed Tyr (63.4%), which disrupted all side-chain/side-chain H-bonds (AO187-Asn ND2/AO232-Asn OD1; AO232-Asn ND2/AO231-His NE2; AO236-Asn ND2/AO232-Asn OD1) and two side-chain/main-chain H-bonds (AO232-Asn ND2/AO228-Ser O and AO236-Asn ND2/AO232-Asn OD1). The Tyr232 only forms one side-chain/main-chain H-bond with Asn236 (AO236-Asn ND2/AO232-Tyr O) (Figures 3I,J).

To comprehensively explore the mutation patterns of *FOXG1*, variants annotated as “pathogenic” and “likely pathogenic” from NCBI ClinVar and DECIPHER were aligned against the CDS of *FOXG1* (Supplementary Figure S2). Totally, 171 mutations in the CDS of *FOXG1* were recruited in both databases. Among 38.60% (66/171) were missense mutations, 37.43% (64/171) were frame-shift mutations, and 23.98% (41/171) were non-sense mutations. For the missense mutations, about 93.94% (62/66) were located in the forkhead domain (FHD) which was responsible for DNA binding, 4.55% (3/66) in the JARID1B binding domain (JBD) responsible for the interaction between FOXG1 and JARID1B (also called as KDM5B). Only one likely-pathogenic missense (c.1439A>G, p.Gln480Arg) was localized in the C-terminal disordered region. As for the pathogenic insertion/deletions (ins/del), they were distributed throughout the whole region of FOXG1.

### Copy number variations containing the *FOXG1* gene

**Figure 4:**
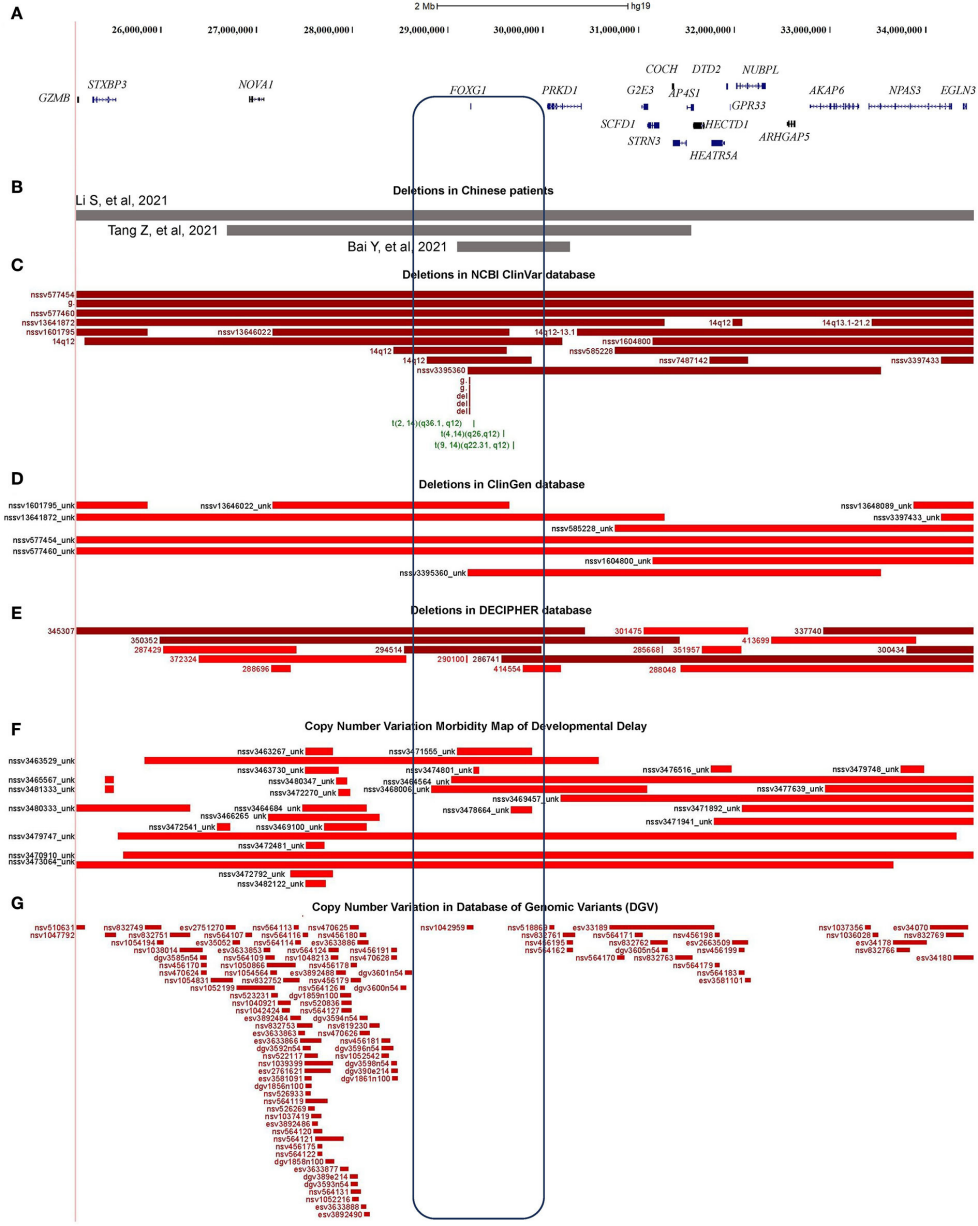
Characterization of the microdeletions containing FOXG1. **(A)** Genomic regions and protein coding genes of microdeletion in patients 9, 10, and 11; **(B)** Covered regions of the two Chinese microdeletions; **(C)** Microdeletions in ClinGen database; **(D)** Microdeletions in NCBI's ClinVar database; **(E)** Microdeletions in DECIPHER database; **(F)** Microdeletions in Developmental Delay; **(G)** Microdeletions in Database of Genomic Variants (DGVs).

Through oligonucleotide array-CGH, four different CNVs were identified, three microdeletions in patients 9, 10, and 11, and one microduplication in patient 12 (Table 1). As for the three microdeletions, the one in Li et al. (2021) was about 9.61 Mb in length (14:25,084,632–34,690,056) and contained 19 protein-encoding genes, including *FOXG1* (Li et al., 2021). Another 4.82 Mb microdeletion (14:26,622,393–31,444,468) in Tang et al. (2021) was completely covered by Li et al. (2021) and contained seven protein-coding genes (such as *NOVA1, FOXG1, PRKD1, G2E3, SCFD1, COCH*, and *STRN3*) (Table 2). The last microdeletion in Bai et al. (2021) was the shortest (14:29,128,665–30,217,058, 1.09Mb) and covered only two genes, such as *FOXG1* and *PRKD1* (Figures 4A,B) (Bai et al., 2021). A 1.90 Mb microduplication at 14q12 (14:27,974,743–29,875,213) was discovered in patient 12 and contained only the *FOXG1* gene (Supplementary Figure S3) (Wang et al., 2017). There were no obvious chromosomal aberrations detected in their parents.

**Table 2:** Haploinsufficiency predictions for the 7 shared genes in microdeletions of three patients.

**Name**	**pHI**	**Inheritance** **model (pA(D)**	**Phenotype/ OMIM**	**Development disorder** **genotype – phenotype** **(DDG2P)**	**Gene curation coalition (GenC(C)**	**ClinGen**
*NOVA1*	2.330	0.977: VLD	/	/	/	/
***FOXG1***	**3.110**	**0.998: VLD**	613454/Rett syndrome, congenital variant	Monoallelic; Loss of function; Congenital Variant of Rett Syndrome;	Definitive: 3 Strong: 1	Definitive: AD Haploinsufficiency: 3 Triplosensitivity: 0
***PRKD1***	**2.790**	**0.698: LD**	617364/Congenital heart defects and ectodermal dysplasia (CHDE**(D)**	Monoallelic; All missense/in frame; Syndromic Congenital Heart Defects	Limited: 1	/
*G2E3*	13.460	0.213: LR	/	/	/	/
*SCFD1*	9.330	0.822: VLD	/	/	/	/
*COCH*	15.090	0.355: LR	601369/Deafness, autosomal dominant 9 (DFNA9) 618094/Deafness, autosomal recessive 110 (DFNB110)	/	Definitive: 1	Definitive: AD
*STRN3*	7.730	0.725: LD	/	/	/	/

VLD, Very likely dominant; LD, Likely dominant; VLR, Very likely recessive; LR, Likely recessive; pAD, probability of being autosomal dominant.

The bold means the two genes, FOXG1 and PRKD1, contained in the shortest microdeletion.

About 76 individual deletions in the genomic region [Li et al. (2021), chr14: 25,084,632–34,690,056] were recruited in different public databases, such as 10 in ClinGen (Clinical Genome Resource), 21 in ClinVar, 16 in DECIPHER, and 29 in the Copy Number Variation Morbidity Map of Developmental Delay (Figures 4C–F) databases. Out of these microdeletions, 31 of them (40.79%) spanned the CDS of the *FOXG1* gene, 5 in ClinGen, 14 in ClinVar, 4 in DECIPHER, and 8 in the Developmental Delay databases. The Database of Genomic Variants (DGV) involving healthy individuals were also checked and only one short microdeletion (nsv1042959) was obtained (Figure 4G). Six short deletions just contained the *FOXG1* gene in which the three shortest deletions, 1057648 (chr14: 29,236,486–29,237,955, 1.47 Kb, ClinVar), 820618 (chr14: 29,236,466–29,237,975, 1.51 Kb, ClinVar), and 290100 (chr14: 29,236,278–29,237,804, 1.52 Kb, DECIPHER), just covered the protein-coding sequence of *FOXG1*.

### Mutation analysis of *PRKD1* gene

**Figure 5:**
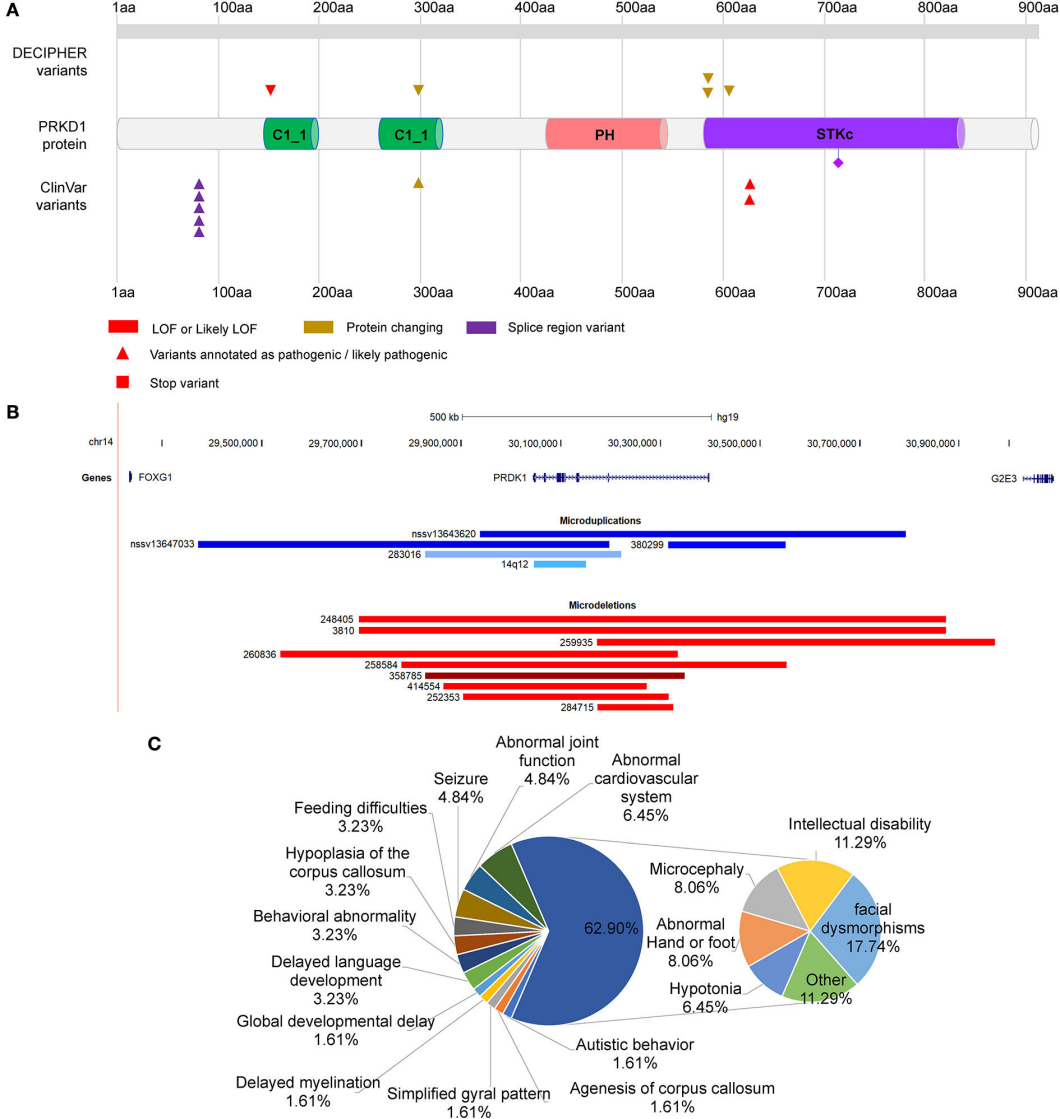
Distribution of mutations in PRKD1. **(A)** Distribution of SNMs of PRKD1; **(B)** Copy number variations of PRKD1; **(C)** Ratios of clinical phenotypes in patients with CNVs containing PRKD1.

*PRKD1* was also contained in the two microdeletions of patients 8 and 9. Therefore, it is necessary to assess the contribution of *PRKD1* to the clinical phenotype of FOXG1-related disorder. After compiling the pathogenic mutations in the CDS of *PRKD1* from published articles, the ClinVar and DECIPHER databases, 13 patients were found to have single nucleotide mutations annotated as pathogenic or likely pathogenic (Figure 5A and Supplementary Table S1). Based on the mode of inheritance of these patients, five were heterozygous (4 *de novo* and 1 unknown), seven were homozygous (from two consanguineous families), and one was unknown. It is worth noting that patients with homozygous mutations, all of them suffered only from non-syndromic congenital heart diseases (CHDs) and no other systemic phenotypes were manifested (Shaheen et al., 2015; Massadeh et al., 2021). The heterozygous mutations could lead to not only CHDs but also abnormalities of the CNS, such as intellectual disability, global developmental delay, hearing impairment, delayed language development, or microcephaly (Swaminathan et al., 2012; Sifrim et al., 2016).

There were also five microduplications and nine microdeletions completely or partially covering only *PRKD1* recruited in the ClinVar and DECIPHER databases (Figure 5B, Supplementary Table S2). Since there were no clinical phenotypes for 4 microduplications (14q12, nssv13647033, and nssv13643620 in ClinVar and 380299 in DECIPHER), 10 CNVs (9 microdeletions and 1 microduplication) were remained for subsequent analysis. Except for abnormalities of the cardiovascular system (Tetralogy of Fallot, Ventricular septal defect, and hypertension) in 6.45% (4/62) of all the reported clinical phenotypes, there also exhibited RTT-like phenotypes, such as facial dysmorphisms (17.74%, 11/62), intellectual disability (11.29%, 7/62), microcephaly (8.06%,5/62), hypotonia (6.45%, 4/62), seizure (4.84%, 3/62), hypoplasia of the corpus callosum (3.23%, 2/62), delayed language development (3.23%, 2/62), behavioral abnormality (3.23%, 2/62), feeding difficulties (3.23%, 2/62), global developmental delay (1.61%, 1/62), agenesis of corpus callosum (1.61%, 1/62), delayed myelination (1.61%, 1/62), simplified gyral pattern (1.61%, 1/62), and others (11.29%, 7/62) (Figure 5C). As for others, it included bruxism, hypothyroidism, intrauterine growth retardation, microdontia, nystagmus, obesity, and short stature. It is very likely that serine/threonine-protein kinase D1 (PRKD1) acts as an independent contributor or collaborator with *FOXG1*, for the clinical phenotypes of a congenital variant form of Rett syndrome.

### Intergenic regulatory elements in the region between *FOXG1* and *PRKD1*

**Figure 6:**
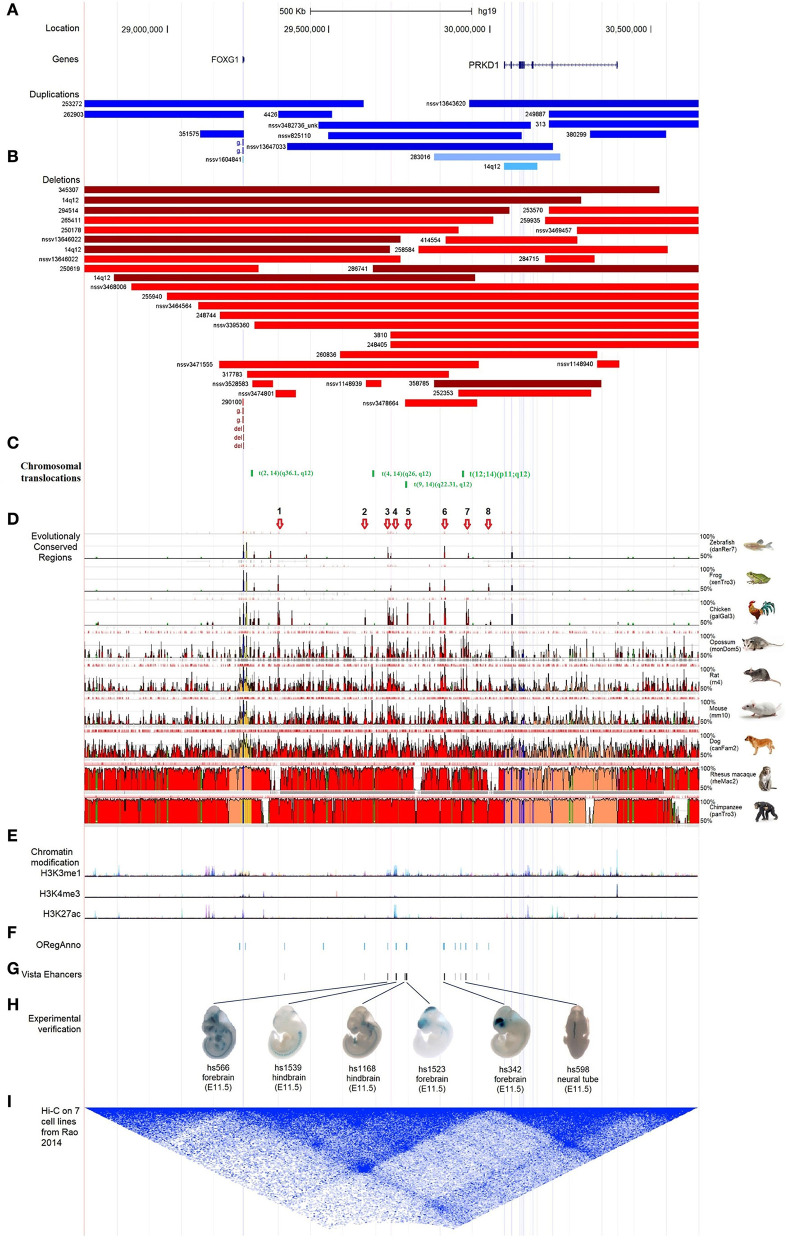
Characterization of the regulatory elements in the intergenic region between FOXG1 and PRKD1. **(A)** Partially-covered microduplications; **(B)** Partially-covered microdeletions; **(C)** Chromosomal translocations; **(D)** Evolutional conservation analysis by ECR browser; **(E)** Chromatin modification of 7 human cells from ENCODE; **(F)** Regulatory elements in ORegAnno database; **(G)** Enhancers in VISTA enhancer browser; **(H)** 6 neuronal active enhancers in mouse embryos; **(I)** In situ Hi-C for seven human cell lines.

According to the reports regarding the large-scale identification of functional elements in the human genome revealed by the Encyclopedia of DNA Elements (ENCODE) Consortium, intergenic non-coding regions often contain multiple regulatory elements, such as enhancers, silencers, or insulators (Maurano et al., 2012). There were many pathogenic CNVs covered the intergenic genomic region between *FOXG1* and *PRKD1* (*FOXG1*-*PRKD1*) (Figures 6A,B). It is implied that the *FOXG1-PRKD1* might be involved in the pathogenesis of FOXG1-related encephalopathy. Interestingly, four *de novo* inter-chromosomal translocations involving the intergenic region *FOXG1*-*PRKD1* were identified in patients with Rett syndrome, congenital variant (OMIM:613454) (Goubau et al., 2013; Mehrjouy et al., 2018), which was also named as FOXG1-related encephalopathy. These translocations were *t*_(2, 14)_ (q36.1, q12), *t*_(4, 14)_ (q26, q12), *t*_(9, 14)_ (q22.31, q12), and *t*_(12, 14)_ (p11, q12) (Figure 6C). The CDS of FOXG1 in these patients was intact and could produce proteins with normal functions, implying that the missing genomic regions might be able to regulate the expression levels of *FOXG1*.

To evaluate the contribution of intergenic CNVs to the phenotypes of *FOXG1*-related encephalopathy, longer CNVs spanning the whole region of *FOXG1* plus *PRKD1* were removed and only those partially covering *FOXG1* or *PRKD1* were left for subsequent analysis. Genomic sequences from zebrafish, *Xenopus tropicalis*, chicken, opossum, rat, mouse, dog, Rhesus macaque, and chimpanzee were compared against the human genome (chr14:28,827,675–30,608,124, hg19), *FOXG1* was strongly conserved during evolution in all the selected animals. As for *PRKD1*, it appeared in mammals and might be a mammalian-specific gene (Figure 6D). Besides, eight strongly evolutionarily conserved regions (SECRs) were identified in the intergenic *FOXG1*-*PRKD1* region (Figure 6D). According to the chromatin modification patterns from ENCODE project, the *FOXG1*-*PRKD1* region might contain several regulatory elements, such as enhancers or silencers (Figure 6E). Fourteen annotated regulatory elements were contained in this genomic region, which was collected in the Open Regulatory Annotation (version 3.0, ORegAnno) database (Lesurf et al., 2016) (Figure 6F). Most of them overlapped with the SECRs. For the annotated regulatory elements, 12 of them (70.59%, 12/14) were tested for enhancer activity in transgenic mouse embryos (E11.5) with LacZ staining in the VISTA enhancer browser (Visel et al., 2007). Six of the SECRs acted as active enhancers, three in the mouse forebrain (hs566, hs1523, and hs342), two in the hindbrain (hs1539 and hs1168), and one in the neural tube (hs598) (Figures 6G,H). After analyzing the *in situ* Hi-C (High-through Chromosome Conformation Capture) data for seven human cell lines (GM12878, K562, KBM7, HMEC, HUVEC, IMR90, and NHEK), this region has three topologically associating domains (TADs). *FOXG1* and the intergenic region (*FOXG1*-*PRKD1*) were completely constrained in the second TAD (Figure 6I), which was completely encompassed by linkage disequilibrium (LD) blocks that were divided by recombination hotspots.

## Discussion

The *FOXG1* (OMIM#164874) is a single exon gene, located in 14q12, which encodes a forkhead transcription repressor. *FOXG1* is expressed highly in the telencephalon, nasal retina, otic vesicles, and olfactory placodes, and serves as a hallmark of the telencephalon in vertebrates (Toresson et al., 1998). It plays a determining role in the development of the telencephalon, cerebral cortex, and genesis of corpus callosum (Manuel et al., 2010). The expression level of *FOXG1* at specific developmental timing is critical for the development of neuronal GABAergic inhibitory circuits. In mouse models, no matter increased or decreased expression of *FOXG1* in both excitatory and inhibitory neurons could be detrimental to the inhibitory circuit formation and result in ASD-like social impairments (Miyoshi et al., 2021). It also plays an important role in the patterning of the early rostral brain and pacing of the telencephalic neurogenesis, specifically stimulating the dendrite elongation (Chiola et al., 2019). Although controlling the neurological development of the telencephalon in the embryonic period, the expression of *FOXG1* continues after birth and through adulthood to prevent the apoptosis and promote the survival of postmitotic neurons (Dastidar et al., 2011), to maintain the neural plasticity (Yu et al., 2019), and to promote the formation of the hippocampal dentate gyrus, especially during early postnatal stage (Tian et al., 2012), which is vital to high-grade function.

In recent years, with the application of molecular genetic testing approaches including chromosomal microarray analysis (CMA), exome array, gene-targeted testing (multigene panel), whole exome sequencing (WES), and whole genome sequencing (WGS), it is found that unlike many other monogenic diseases with clear and single mutation sites and mutation modes, the genomic region of the *FOXG1* gene is very unstable, resulting in diversified mutation types. The reported cases carried dozens of mutation types, such as non-sense, missense, frameshift, initiator loss, terminator loss, large fragment duplication, and large fragment deletion. These mutations were distributed in different functional regions of *FOXG1* (Wong et al., 2019b). Since the *FOXG1* gene contains only one exon, the resultant non-sense and frameshifting transcripts could be translated into aberrant proteins, instead of being degraded by the non-sense-mediated mRNA decay (NMD) (Kurosaki et al., 2019). The diversity of mutation sites and mutation types could affect the brain developmental events regulated by FOXG1 to varying degrees, which eventually led to the diversity of clinical manifestations of FOXG1-related encephalopathies.

Currently, there were four Chinese patients identified to have heterozygous large fragment abnormalities in their genomes, three microdeletions, and one microduplication. In order to find out if there existed other 14q12 copy number variants (CNVs), large-scale CNV screening analyses for Chinese patients with developmental delay/intellectual disability (DD/ID) were reviewed. Totally, in 2,870 DD/ID complied cases, 707 pathologic/likely pathologic CNVs were identified, accounting for 24.86% of the patients. However, no more 14q12 CNVs involving FOXG1 were identified. It is indicated that the occurrence of FOXG1-related disorder was extremely rare in China. The minimally overlapped region of the three microdeletions contains two protein-coding genes (*FOXG1* and *PRKD1*). *FOXG1* and *PRKD1* were reported to cause the occurrence of a congenital variant of Rett syndrome (OMIM#613454) and congenital heart defects and ectodermal dysplasia (CHDED) (OMIM#617364), respectively. In the ClinGen database, *FOXG1* was curated as a haploinsufficient gene with dosage pathogenicity. Although no annotations about *PRKD1* in the ClinGen database, it has many similar features to *FOXG1*, such as being haploinsufficient, dominantly inherited, and contained in the same microdeletions or microduplications with *FOXG1*, it is very difficult to exclude *PRKD1* from the underlying genetic factors for FOXG1-related disorder.

After compiling CNVs recruited in several databases, such as ClinGen, ClinVar, and DECIPHER, six shortest CNVs just covered the CDS of *FOXG1*, four microdeletions (nssv3442672 in ClinGen, 1073841 and 830776 in ClinVar, and 290100 in DECIPHER) and two microduplications (1020268 and 471463 in ClinVar). It has been reported that *FOXG1* was vital for the telencephalon development, the survival of postmitotic neurons, neural plasticity, and the formation of the hippocampal dentate gyrus, therefore, *FOXG1* should be the crucial molecular factor for disorders with 14q12 abnormalities.

The *PRKD1* gene was known as the molecular etiology for CHDED (Sifrim et al., 2016). In the case of patients with heterozygous mutations in the CDS of the *PRKD1* gene, they suffered from not only CHDs but also intellectual disability, global developmental delay, hearing impairment, delayed language development, or microcephaly (Swaminathan et al., 2012; Sifrim et al., 2016). These clinical features were very similar to those of FOXG1-related disorders. To further understand the possibility of *PRKD1* to FOXG1-related disorder, clinical features were compiled from patients with deletions or duplications containing only the *PRKD1* gene. As for the one microduplication and nine microdeletions covering only the *PRKD1* gene with detailed clinical phenotypes in the ClinVar and DECIPHER databases (Figure 5B, Supplementary Table S2), in addition to abnormalities of the cardiovascular system, clinical features similar to FOXG1-related encephalopathy were also identified. The encoded protein PRKD1 is a serine/threonine protein kinase involved in many cellular processes, such as Golgi body membrane integrity and transport, cell migration and differentiation, and cell survival. It is important for neuronal polarity, synapse formation, and synaptic plasticity (Bisbal et al., 2008; Yin et al., 2008; Cen et al., 2018). PRKD1 also plays an important anti-apoptotic survival role for dopaminergic neurons during the early stage of oxidative stress (Asaithambi et al., 2011). It had been reported that *PRDK1* was associated with intelligence (Hill et al., 2019), cognitive performance (Lee et al., 2018), depressive symptoms (Baselmans et al., 2019), and susceptibility to schizophrenia (a kind of psychiatric disorder) (Pantelis et al., 2014; Wu et al., 2020). Overall, *PRKD1* might be a contributing factor for the varying clinical phenotypes of FOXG1-related encephalopathies.

**Figure 7:**
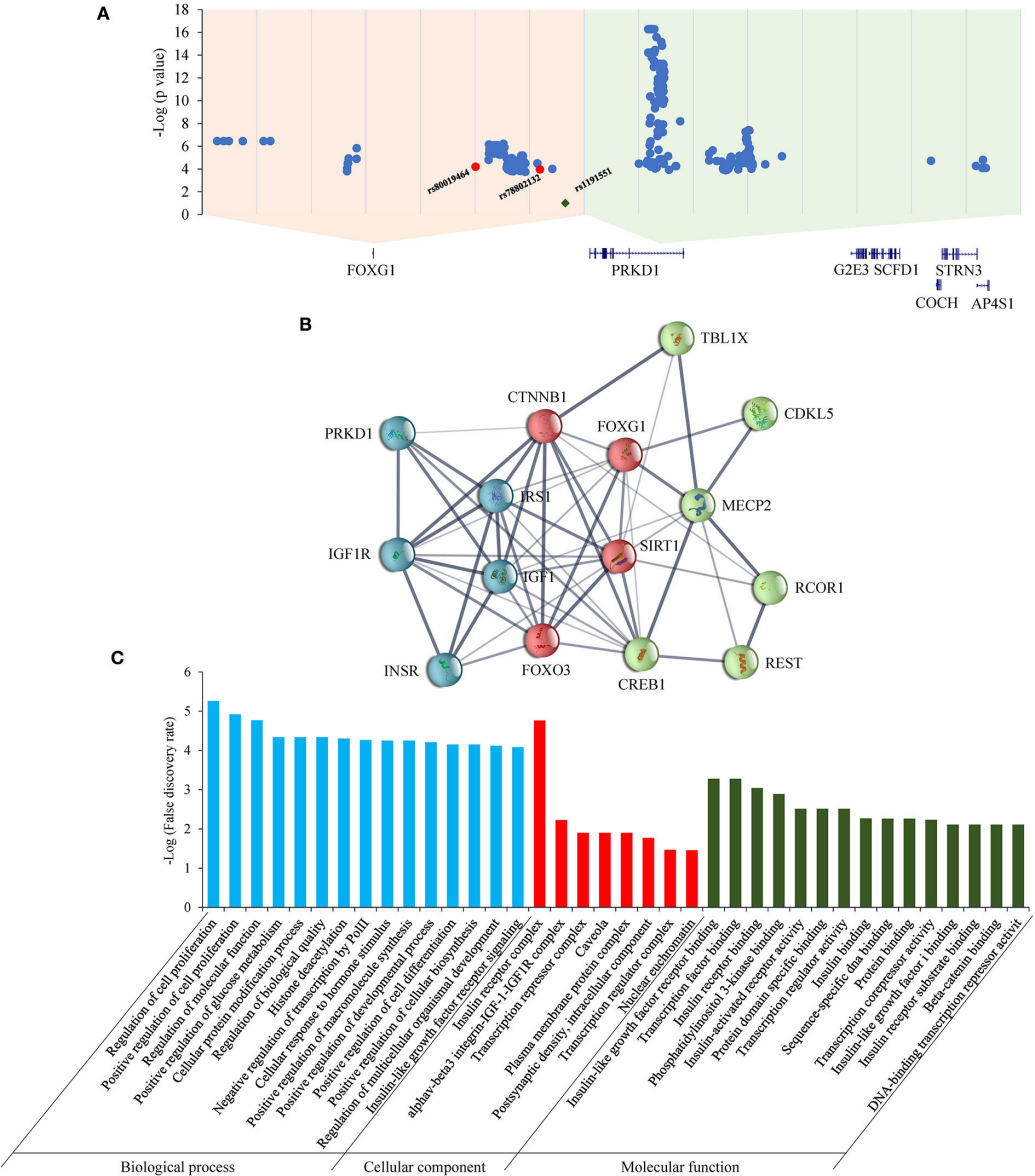
eQTLs and STRINGed network analysis for FOXG1 and PRKD1. **(A)** eQTLs for FOXG1 and PRKD1; **(B)** STRINGed protein interaction network. **(C)** GO analysis for the interacting components.

As for the *FOXG1-PRKD1* intergenic region, most of the CNVs overlapped partially or completely with the intergenic region (Figure 6), it is inferred that the *FOXG1–PRKD1* region might play some unknown functions for the disease. Six of the SECRs in the intergenic region were experimentally verified as active enhancers in the embryonic mouse brain (Visel et al., 2007). Based on the *in situ* Hi-C data for human cells (Rao et al., 2014), *FOXG1* and the *FOXG1-PRKD1* region were completely contained in a large topologically associating domain (TAD). In this TAD, a schizophrenia-related SNP, rs1191551 was localized in close vicinity to the last putative enhancer (element_555), which was 760 kb away from the gene body of *FOXG*1. Reporter assay and genomic editing by CRISPR/Cas9 showed that the short region containing rs1191551 regulated the expression of *FOXG1* but not the nearby *PRKD1* (Won et al., 2016). According to the significant single tissue expression quantitative trait loci (eQTLs) for *FOXG1* and *PRKD1* curated in the Genotype-Tissue Expression (GTEx) project, there were 137 eQTLs that were exclusively located in the FOXG1-specific TAD. As for the 214 eQTLs of *PRKD1*, only two (rs80019464 and rs78802132, 0.93%) were located in the FOXG1-specific TAD (Figure 7A). These imply that the intergenic regulatory elements primarily regulate the expression of *FOXG1*, instead of *PRKD1*, by a cis-acting model. However, it is not clear about the plethora of transcription factors and regulatory manners of the intergenic enhancers to control the expression of FOXG1.

The STRINGed network for the 14 proteins involving well-known RTT or RTT-like genes, such as MECP2, CDKL5, and FOXG1, plus PRKD1 was constructed using Cytoscape (version 3.9.0) (Figure 7B). FOXG1 could interact with MECP2 and CDKL5. IGF1-IGF1R complex and INSR-IRS1 complex could regulate directly the expression of FOXG1 and indirectly the expression of MECP2 and CKDL5. As for PRKD1, it had been reported that PRKD1 could interact with IGF1R (Hermanto et al., 2002). Gene Ontology (GO) analysis revealed that genes in the GO term “regulation of cell proliferation” in the biological process were the most significantly enriched (*p* = 5.54E-06) (Figure 7C). Genes in the “insulin receptor complex” in cellular component and “IGF1R binding” were the second and third most enriched. It implied that insulin or insulin-like factor pathways might play an important role in the pathogenesis of RTT or RTT-like syndrome. It has been reported that IGF1 could ameliorate RTT-relevant phenotypes in animal models and improve some clinical manifestations in clinical trials (Pini et al., 2012; Keogh et al., 2020). Since possessing the ability to bind and regulate the expression of IGF1R, it is very likely that *PRKD1* is a novel contributor to the clinical phenotypes of FOXG1-related disorder. Experiments under cellular and animal levels should be carried out to provide solid evidence showing the involvement of PRKD1 in the pathogenesis of FOXG1-related encephalopathy.

## Conclusion

Based on our comprehensive reanalysis of FOXG1 mutations, the molecular etiologies for FOXG1-related encephalopathies were quite complex. It could result from mutations in the CDS of FOXG1 itself, microdeletion/microduplication of the whole FOXG1, microdeletion/microduplication of the regulatory elements in the intergenic *FOXG1-PRKD1* region, and modified by PRKD1. The management of FOXG1-related encephalopathy is a great challenge for medical practitioners.

## Data availability statement

The datasets presented in this study can be found in online repositories. The names of the repository/repositories and accession number(s) can be found in the article/Supplementary material.

## Ethics statement

The studies involving human participants were reviewed and approved by Shenzhen Baoan Women's and Children's Hospital. Written informed consent to participate in this study was provided by the participants' legal guardian/next of kin. The animal study was reviewed and approved by Shenzhen Baoan Women's and Children's Hospital. Written informed consent was obtained from the individual(s), and minor(s)' legal guardian/next of kin, for the publication of any potentially identifiable images or data included in this article.

## Author contributions

GL and JW conceived the project, wrote, and revised the manuscript. QP and YZ performed genetic counseling, analyzed the clinical data, and revised the manuscript. HX and XH performed the bibliographic search and analyzed the data. PX, LW, and DL collected data and recruited mutations. All authors critically revised the article for important intellectual content.
