# Supplementary material for: Identification of a de novo mutation of the FOXG1 gene and comprehensive analysis for molecular factors in Chinese FOXG1-related encephalopathies
Source: Front Mol Neurosci. 2022 Dec 7;15:1039990. doi: 10.3389/fnmol.2022.1039990 (PMC9768341; doi:10.3389/fnmol.2022.1039990)
Supplement: Supplementary file 3 [file Data_Sheet_1.docx]

# Supplementary Figures

## Figure S1.


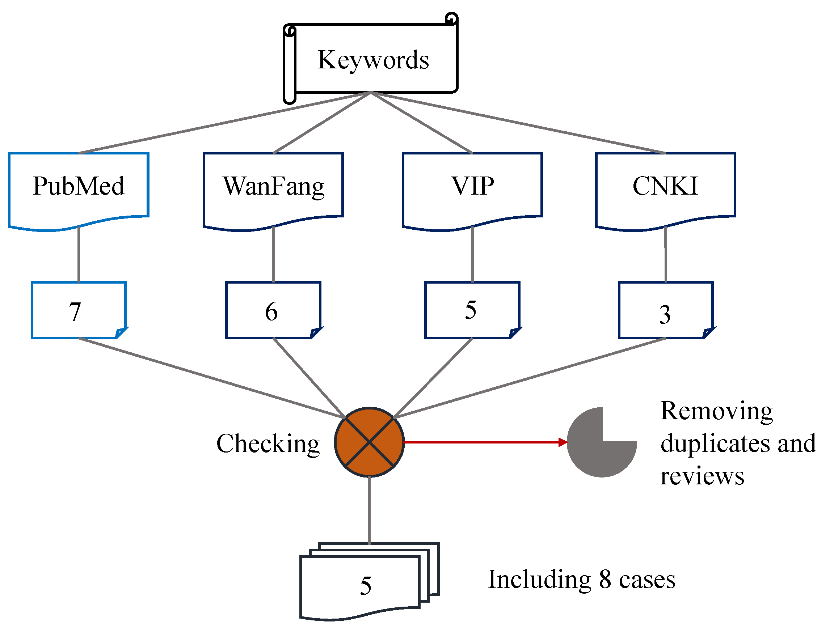


**Figure S1**. **Workflow chart of data mining.**

## Figure S2.


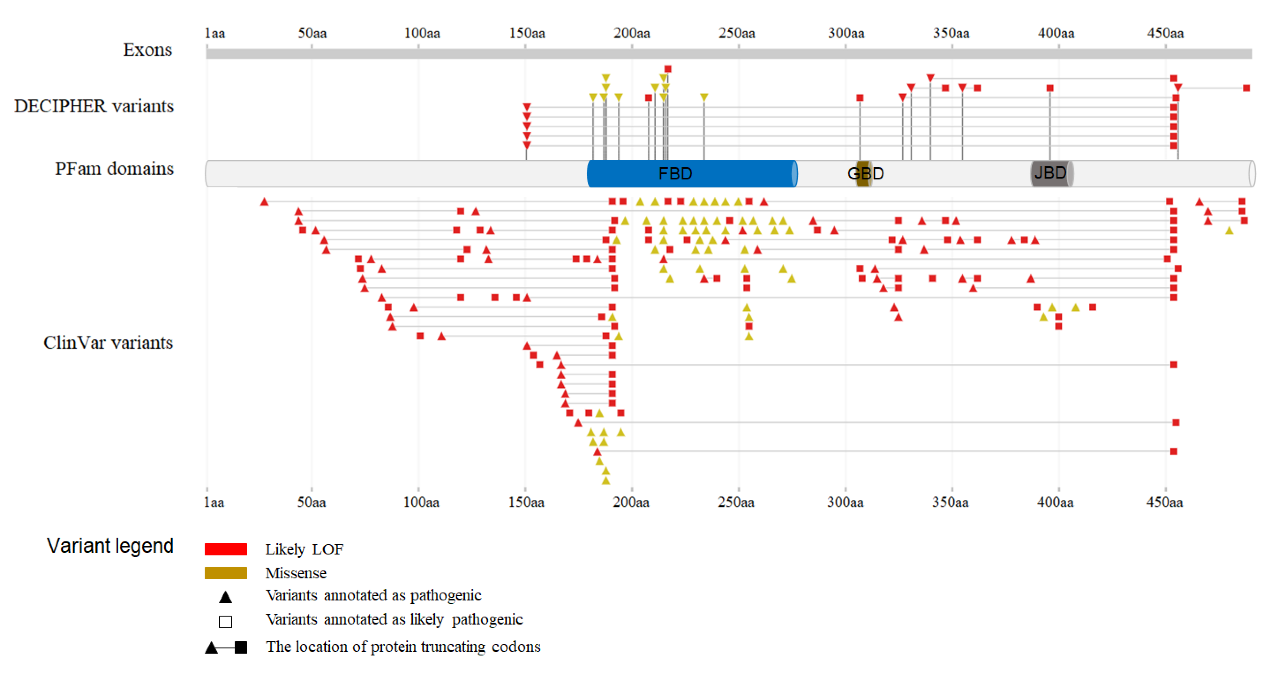


**Figure S2. Identified mutations of FOXG1 in DECIPHER and ClinVar databases.**

## Figure S3.


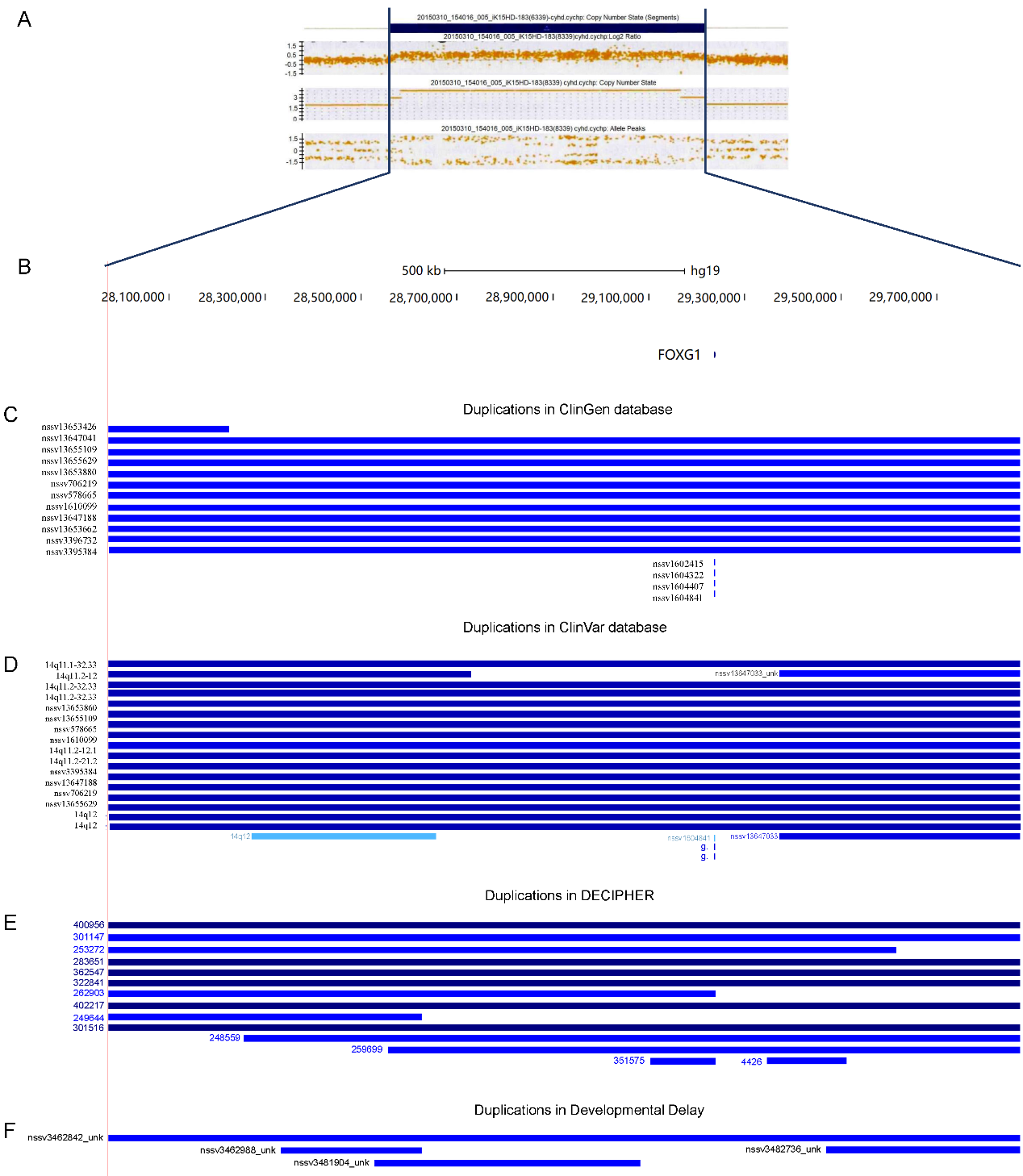


**Figure S3**. **Microduplication covering 14q12(27974743-29875213)**. A) Chromosomal microarray analysis for patient 7; B) Genomic region; C) Microduplications in ClinGen database; D) Microduplications in ClinVar database; E) Microduplications in DECIPHER database; F) Microduplications in Developmental Delay data.
